# Film Control to Study Contributions of Waves to Droplet Impact Dynamics on Thin Flowing Liquid Films

**DOI:** 10.3791/57865

**Published:** 2018-08-18

**Authors:** Idris T. Adebayo, Omar K. Matar

**Affiliations:** ^1^Department of Chemical Engineering, Imperial College London

**Keywords:** Engineering, Issue 138, Film control, droplet impact, flowing films, waves, bouncing, coalescence, splashing, high-speed imaging

## Abstract

Droplet impact is a very common phenomenon in nature and attracts attention due to its aesthetic fascination and wide-ranging applications. Previous studies on flowing liquid films have neglected the contributions of spatial structures of waves to the impact outcome, while this has recently been shown to have a significant influence on the drop impact dynamics. In this report, we outline a step-by-step procedure to investigate the effect of periodic inlet forcing of a flowing liquid film leading to the production of spatiotemporally regular wave structures on drop impact dynamics. A function generator in connection with a solenoid valve is used to excite these spatiotemporally regular wave structures on the film surface while the impact dynamics of uniform-sized droplets are captured using a high-speed camera. Three distinct regions are then studied; *viz.* the capillary wave region preceding the large wave peak, the flat film region, and the wave hump region. The effects of important dimensionless quantities such as film Reynolds, drop Weber and Ohnesorge numbers parameterized by the film flow rate, drop speed, and drop size are also examined. Our results show interesting, hitherto undiscovered dynamics brought about by this application of film inlet forcing of the flowing film for both low and high inertia drops.

**Figure:**
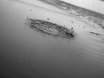


## Introduction

Droplet impact is a very common phenomenon in nature and attracts attention from any curious observer^1^. It constitutes an active research area due to its numerous applications including spray-cooling, fire-suppression, inkjet-printing, spray-coating, deposition of solder bumps on printed circuit boards, the design of internal combustion engines, surface-cleaning, and cell-printing^2^. Its application extends also to agriculture, *e.g., *sprinkling irrigation and crop spraying^3^,^4^. Pioneering work dates back to the 19^th^ century, with the work of Worthington^5^, while major advances have only been recently made due to the emergence of high-speed imaging^6^. Since then, several studies have been carried out; using different types of impact surfaces ranging from solids^7^,^8^, shallow,^9^ and deep liquid pools^10^,^11^ to thin films^12^,^13^.

However, despite the large volume of research on droplet impact on liquid surfaces (*i.e.,* shallow and deep pools and quiescent films), impact on flowing thin liquid films has not received as much attention. In addition, hitherto, studies have neglected the contributions of spatial structures of waves to droplet impact dynamics.

In this report, we present a detailed experimental procedure to investigate the droplet impact process on flowing films whose dynamics are influenced by inlet-forcing of the liquid flow rate; below, we refer to them as 'controlled' films. We find that these have numerous applications in multiphase industries (*e.g., *in cooling towers, in distillation columns, and also in the annular flow regime observed in two-phase flows), especially as film control has become an important step in the intensification of both heat and mass transfer in many process industries^14^. The interested reader is referred to our previous work^15^ for more details on the results of our research efforts on this.

This application of frequency oscillations of the inlet flow rate results in the formation of regular waves on the film surface. We focus on the solitary wave family, which is essentially characterized by widely-separated narrow peaks and is preceded by a series of front-running capillary waves^16^,^17^,^18^. We study the outcome of the impacts associated with the three main parts of the solitary wave structure: the 'flat film', 'wave hump', and front-running 'capillary wave' regions. We also contrast these results with those associated with uncontrolled flowing films. Our results show that the stochastic nature of wave appearance on the uncontrolled film markedly affects the outcome of drop impact, with the separate regions of the controlled film also showing new mechanisms, which we have detailed both qualitatively and quantitatively.

In the previous paper^15^, using the same procedure, we studied the effect of film control on droplet impact dynamics in the splashing regime. The obtained results showed both quantitative and qualitative differences in the crown morphology (height, diameter, wall thickness, tilt angle, and direction) as well as the number and size distribution of the ejected secondary droplets.

In this report, we describe the designed set-up to understand the critical role played by these spatial structures in the droplet impact dynamics and also present succinct details of our findings not only in the splashing regime but also for other outcomes of droplet impact (*viz.* bouncing, sliding, partial/total coalescence). By following the standard protocol described below, the effect of film control on the droplet impact dynamics can be studied in a reproducible fashion.

## Protocol

### 1. Experimental Rig Setup

NOTE: See Figure 1.

1. **Falling film unit**
  1. Start by cleaning the substrate (glass) surface with a clean, soft cloth. Ensure no dirt is adhered to its surface, which would alter the liquid properties.
  2. Set the pivot of the glass substrate to the desired inclination angle. An inclination angle, β, of 15˚ was used in this work.
  3. Switch on the electric pump and ensure a normal liquid flow on the film surface to further clean the glass substrate. For this work, the test liquid was deionized water.
  4. Ensure the entire surface of the substrate is wetted.
  5. Measure the film flow rate using the flow meter. For this work, the flow rate was varied between 1.667 x 10^-3^ and 10 x 10^-3^ m^3^/s with the corresponding film Reynolds number, *Re = ρq*/*wµ*, ranging between 55.5 and 333. *w* is the falling film width, 0.30 m.
  6. Gradually adjust the valves on the flow connection to obtain the desired flow rate on the glass substrate.
  7. Adjust the set of micrometer step at the film inlet to the corresponding Nusselt film thickness value for the chosen flow rate, to avoid a hydraulic jump at the film inlet or a backflow of air into the distribution chamber.
  8. Manually siphon all air in the distribution chamber to obtain a uniform flow downstream on the film surface.
2. **Film control unit**
  1. Ensure that the function generator is connected to the solenoid valve through a non-latching relay via a data acquisition card (DAC).
  2. Switch on both the solenoid valve and the function generator.
  3. Set the function generator to the desired forcing frequency. In this work, frequencies of 2 and 3 Hz were used.
  4. Choose the desired wave signal (sine wave, sawtooth wave, square wave, *etc.*). In this work, a sine wave signal was used. Figure 2A and **2B** show the contrast between an uncontrolled film and a controlled film.
3. **Droplet generation system**
  1. Attach a clean plastic tubing to a water-filled syringe.
  2. Insert the syringe into the droplet generator.
  3. Affix a syringe-needle of a chosen size (depending on the desired droplet diameter) to the other end of the plastic tubing. The droplet diameter range studied was between 0.0023 to 0.0044 m.
  4. Adjust the fall height of the drop above the film surface. In this work, the drop's fall height was varied from 0.005 to 0.45 m, giving impact speed between 0.30 ± 0.02 - 2.96 ± 0.06 m/s.
  5. Similarly, set the streamwise impact point of the drop from the film inlet. This was set to 0.3 m in this work to ensure the waves are well-formed prior to impact.
  6. Set a desired flow rate for the syringe pump.
  7. Adjust the flow rate to achieve a droplet generation frequency greater than the wavelength of the waves formed on the film surface; to ensure drops successively impinge on different regions of the controlled film. See Figure 2C; with an enlargement of a singular waveform in Figure 2D to show the dissimilarities in the flow profile beneath each region^19^,^20^.
4. **High-speed imaging setup**
  1. Place the camera on a tripod stand (or any other suitable arrangement).
  2. Select the macro lens with desired focal length and connect this to the camera.
  3. Switch on the high-speed camera and ensure direct focus on the film surface. Align the camera at 7˚ and 12˚ horizontal and vertical deviations respectively to the film surface. This gives an excellent side-view image of the impact process, resulting in a resolution of 67.5 µm/pixel and 46.6 µm/pixel in the streamwise and spanwise directions, respectively.
  4. Adjust the focus of the camera-lens (at the largest aperture) using a calibration item placed exactly on the droplet impact spot.
  5. Once a sharp focus has been obtained, reduce the aperture to ensure only a small amount of light enters the camera.
  6. Set the desired frame rate, resolution, and shutter speed of the high-speed camera. A frame rate of 5000 fps, 800 x 600 resolution, aperture size 1/16, and a shutter speed of 1 µs were used in this work.
  7. Place the light diffuser in front of the light source, as shown in Figure 1C, to ensure the light is uniformly diffused across the imaging region.
  8. Power on the light source to confirm the uniform spreading of light over the imaging area.

### 2. Calibration

NOTE: See Figure 3.

1. Put a ruler in the film flow direction (exactly on the spot of impact) and obtain snapshots of measured points on the film surface.
2. Repeat 2.1 but with the ruler in the spanwise direction.
3. Use the above to obtain the spatial resolutions on the film surface.

### 3. Video Recording and Data Acquisition

1. Once film flow is established on the rig, start the syringe pump and observe the impact of the dripping drops on the film surface.
2. Start the function generator and observe the production of spatiotemporally regular waves on the film surface.
3. Ensure successive drops are impacting the different regions of the controlled film surface.
4. Observe the post-triggering frame number and set this to approximately half of the video length to adequately capture the impact.
5. Power on the light source and trigger the image capturing once an impact occurs.
6. Power off the light source once image capturing is complete to avoid overheating of the liquid film.
7. Visually analyze the obtained snapshot on the computer screen. Check to see if the impact has occurred on one of the flat film, capillary wave, or wave hump regions.
8. Trim down the video to the portion showing the impact process and save the frame range in a video/image format.
9. Repeat 3.5 - 3.8 and record individual impact on all regions on the film surface, viz*.* solitary hump, capillary waves, and flat film.

### 4. Image Post-processing and Analysis

1. Place a ruler in the field of view and calculate the spatial resolution by counting how many pixels fit across 1 cm. Using the calibration image, obtain a scale factor for image dimension measurement.
2. Compare the outcomes of the impact process on the different impact regions from the high-speed images. Check to see notable differences.
3. Using a suitable MATLAB image-processing routine, measure the characteristics features of the product of the impact process: viz. in the splashing mode, measure the crown height, diameter, wall thickness, tilt angle, crown-facing direction, number and size distribution of ejected secondary droplets.
4. Carry out similar quantitative analyses as 4.3 above for the low-Weber impacts. Count the pinch-off time of satellite drops from the time-framed images and measure the apex length and width of the column formed in partial coalescence before pinch-off of secondary drops. Measure the size of ejected secondary drops. Count the number of cascade in a repeated pinch-off process.
5. Observe all qualitative differences in each region.

## Representative Results

Essentially, two categories of impacts were studied; the first was for drops with low inertia (*i.e.,* drop Weber number, (*We_d_*_= _*ρdu*^2^/*σ*) ranging from 3.1 to 24.0 while the second was for drops with high inertia (*i.e.,We_d_* 94 to 539) resulting in a splash outcome. The same experimental procedure, however, was followed for both studies. Other related dimensionless quantities used in the study include the film Reynolds number (*Re *= *ρq*/*wµ*, ranging between 55.5 and 333), the film Weber number (*We *= *ρh_N_u_N_*^2^/*σ*, ranging between 0.1061 and 2.1024), the drop Ohnesorge number (*Oh *= *µ*/(*ρσd*)^1/2^, ranging between 0.0018 and 0.0025) and the Kapitza number (*Ka *= *σρ*^1/3^/g^1/3^*µ*^4/3^, which was calculated to be 3363 for water). The Nusselt film thickness (*h_N_* = [(3*µ*^2^*Re*)*/*(*ρ*^2^gsin*β*)]^1/3^) was found to range from 4.034 x 10^-4^ to 7.328 x 10^-4^ m, while the Nusselt film velocity (*u_N_* = *ρ*gsin*βh_N_*^2^/3*µ*) was found to range from 0.1376 to 0.4545 m/s. For all above equations, *q *is the film flow rate, varying between 0.001667 and 0.01 m^3^/s; *β* is the substrate inclination angle, fixed at 15˚ to the horizontal; *µ *and* ρ* are the viscosity and density, respectively, of water estimated at 0.001 Pa s and 1000 kg/m^3^; *σ* is the surface tension force (0.072 N/m); and g is the gravitational force (9.81 m/s^2^).

In the low inertia impacts, the trends observed, though a little similar (Figure 4), exhibited a number of distinctly spottable differences. First, it was generally noticed that the size of the satellite drop produced on the wave hump region was always bigger compared to other regions of impact. In retrospect, the opposite was found true on the capillary wave region. The satellite drops were always very small. This occurs because the radial wave produced by the impacting drop becomes suppressed by the existent capillary ripples. As a result, further wave propagation to vertically elongate the drop is inhibited, which results in the drop losing its potential to develop a sufficiently long vertical column, thereby leading to the ejection of only tiny secondary drops from the slender columns formed. It was also observed that the tendency of a cascade was much reduced on the wave hump compared to other regions. In all the cases examined, the product of partial coalescence, hardly experienced another partial coalescence, while on a flat film, up to three to four are observed. The column height was also observed to be higher and most tilted in the flow direction on the wave hump region in comparison with other regions.

On the flat film region in comparison with other regions of impact, there is an increase in the tendency of a bouncing outcome. This occurs due to the strong lubrication force exerted on the drop by this thin flat film, which slows down the drainage/thinning of the intervening air layer between the drop and the film, thereby preventing the merger. This then results in the observed drop deformation as well as the eventual lift-off. In comparison, impacts on the wave hump are more prone to partial coalescence, partly due to the thickness of the film, the absence of pre-existing waves (as found in the capillary wave region), and finally the reduced lubrication force caused by flow recirculation in this region. These cumulatively result in the generation of rather longer columns than those produced on other regions.

With an increase in liquid film flow rate (*i.e.,* film *Re*); impacts on the capillary waves often resulted in a gentle sliding of the drop of the capillary wave without merger (see Figure 5a**-5h**). This rolling drop (Figure 5d**-5f**) later then climbs the on-coming solitary hump (Figure 5g** and 5h**) where it experiences a partial coalescence (not shown). However, the outcome of impact on the flat film region changes from a steady partial coalescence to favor the bouncing mode. In the case of the impact on the capillary wave, the increase in film *Re* led to more closely peaked capillary waves which then acted as a "cushion" on which the drop "rode", hence the observed sliding of the drops. At the least *Re*, a very quick pinching off of drop is usually observed on the flat film region (of size 90% of the initial drop), with this drop experiencing some "dancing" mode before it later merges and results in a normal partial coalescence. This is, however, not observed on other regions of the controlled film.

With an increase in drop *We_d_*, it was observed that the column height increased both on the flat film region and the wave hump but reduced on the capillary wave region.

Finally, with an increase in drop size, longer and wider columns were observed on the flat film region, which in turn gave rise to a bigger satellite drop. However, on the wave hump, this was not observed, instead, a transition to total coalescence was observed. On the capillary wave, the increase in drop size led to reduced sliding of the drop and a transition to partial coalescence. The biggest drop, however, yielded almost immediately to total coalescence. A summary of these results is presented in **Table 1**.

Beyond droplet velocity 1.70 ± 0.03 m/s, a splash outcome is observed in all three regions on the film surface (Figure 6). However, though a similar outcome is observed as well in this regime, striking differences are observed in the morphology of crown formed-its height, diameter, wall thickness, tilt angle, coalescing time as well as number and size-distribution of ejected secondary droplets.

In the 'wave hump region', the crown structure is different from that in the 'capillary' and 'flat film regions', as its shape is more regular. It also possesses a thicker crown wall and the crown height is higher than those observed in the 'capillary' and 'flat film regions'. There are also fewer secondary droplets ejected from its rim in comparison with the crowns formed in the other regions. Finally, a longer coalescence time is observed before the crown is swept away by the flowing film.

In the 'capillary wave' and 'flat film region', the crowns formed are also quite different based on a number of features. First, it was observed that the rear height of the crown is affected by the capillary humps as well as the flow reversal dynamics in this 'capillary wave region', hence causing the crown formed to appear more upright. This flow reversal results in the transport of liquid mass backward which augments the rear height of the crown formed. This, however, is not observed on the flat films: the crown is naturally tilted in the liquid flow direction and tilts even further with increasing *Re*. This tilt can be observed in both the upstream and downstream ends of the crown. In comparison, on the capillary waves, as the film *Re* is increased, the rear side of the crown appears to become more 'upright' in a manner quite opposite to that observed on flat films. The crown height on the flat film is however, higher than that on the capillary waves due to the confinement of the substrate. There is also a more rapid onset of secondary droplet ejection from the crown rim, on the capillary waves in comparison with that on flat films. Finally, more secondary droplets are ejected on the rim of the crown on flat films than that on capillary waves*.*

Temporal evolution of the crown shows a weak dependence of the crown diameter on film *Re* in all regions of the flow. The weakest dependence on *Re* is observed in the 'wave hump region'. In the 'flat film region', the crown height is observed to increase with *Re* as expected, since larger *Re *are associated with thicker films. The degree of crown inclination towards the flow direction is also higher with increasing *Re* in the 'flat film', and 'wave hump' regions; this effect, however, appears to be less pronounced in the 'capillary wave region'.

In the 'wave hump region', there are fewer secondary droplets ejected with increasing *Re. *There appears to be a somewhat weak dependence of the crown height on *Re*, while there is a decrease in the crown coalescing time with increasing *Re*, which is the result of the increased speed of the flowing film upon which the impact occurs, which quickly sweeps the coalescing crown away from the original impact point. There is also a change in the inclination of the crown in the 'wave hump region' depending on the competition between the inertia of the impacting drop and that of the flowing film. At lower *Re*, the crown faces the downstream direction, while at higher *Re* values, it faces the upstream (Figure 7). This trend is not observed in the 'capillary wave' and 'flat film regions'.

In the 'capillary wave region', more secondary droplets are observed at lower *Re*. There is also an increase in the overall crown height with *Re*, and, at lower *Re*, droplet ejection is mainly towards the streamwise direction (with the crown rim higher at the front than at the rear and also tilted more towards the streamwise direction). The height becomes more symmetric at higher *Re*, which is believed to be as a result of the balancing effect of the higher humps which capillary waves possess at their rear, thereby balancing-off the crown rim height at the back.

With the drop Weber effect, it can be observed that the crown diameter increases at a greater rate with increasing *We_d_*; the largest rate is associated with the 'wave hump region'. Further differences observed in the number and size distribution of the ejected secondary droplet in this splashing regime are shown in Figure 8 and Figure 9, respectively. A summary of these results is presented in **Table 2**.

**Figure:**
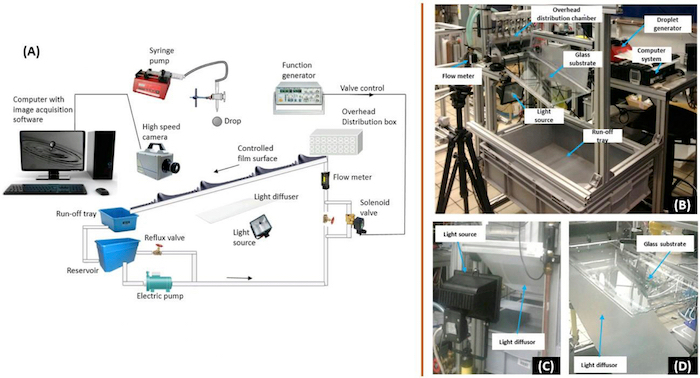

**Figure 1: Experimental rig. (A)** Schematic representation of the experimental rig**, **consisting of the falling film unit for the flow of liquid film on an inclined glass substrate; a film control unit (consisting of a solenoid valve connected across a non-latching relay via data acquisition card and a function generator which sends automated signal controlling the opening and closing of the solenoid valve); a syringe pump used for the generation of droplets of controlled sizes from calculated heights above the film surface, and a high-speed camera for digital imaging. The obtained results are analyzed on the computer system. Reproduced from Adebayo & Matar 2017^15^ by permission of The Royal Society of Chemistry. **(B) **A pictorial view of the rig.** (C) - (D)** Pictorial description of lighting arrangement. Please click here to view a larger version of this figure.

**Figure:**
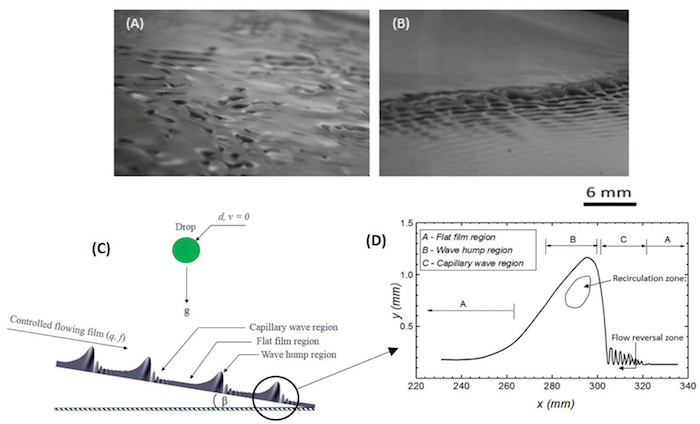

**Figure 2: Effect of film control on wave evolution dynamics on a flowing liquid film.(A)** Shadowgraph image of film surface prior to film control. The film is characterized by the presence of naturally evolving waves which are stochastic in nature and exhibit irregular spatiotemporal dynamics. **(B)** Shadowgraph image of the film surface after forcing. The waves are spatiotemporally regular and predictable, rendering contributions from the spatial structure to drop impact easy to study. **(C)** Solitary wave formation on a controlled flowing liquid film highlighting the different regions on the film surface *viz.* capillary wave, flat film and wave hump regions. **(D)** Magnified view of a singular wave structure showing the flow profile in each zone. Reproduced from Adebayo & Matar 2017^15^ by permission of The Royal Society of Chemistry. Please click here to view a larger version of this figure.

**Figure:**
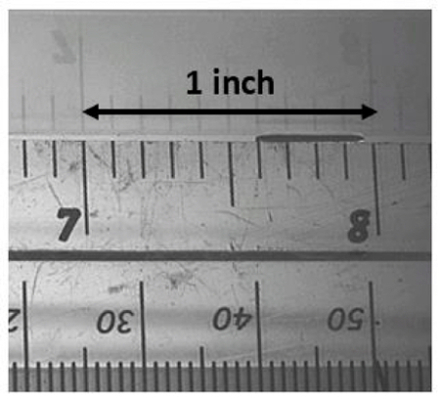

**Figure 3: Spatial resolution at 5000 fps.** With a substrate inclination angle of 15˚, the spatial resolution is calculated to be 67.5 µm/pixel and 46.6 µm/pixel in the streamwise and spanwise directions, respectively. Please click here to view a larger version of this figure.

**Figure:**
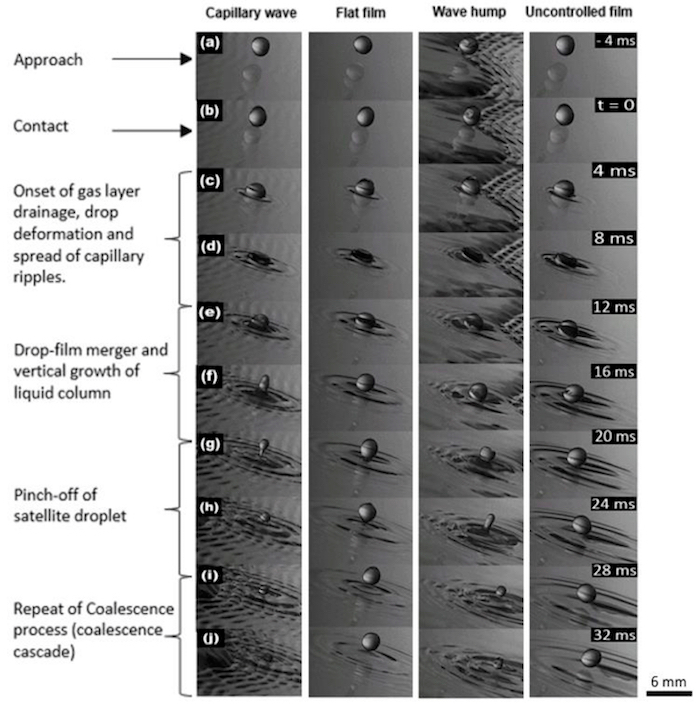

**Figure 4: Effect of film control on the outcome of low inertia drops impacting different regions of a controlled flowing film, contrasted against an uncontrolled film. **The droplet fall-height is 0.005 m, drop size is 3.3 mm, film speed is 5 x 10^-3^ m^3^/s, forcing frequency is 2 Hz, corresponding to film *Re* 166.5, drop *We* 3.134 and *Oh* 0.0021. The drop approaches the film surface (a) and on contact (b), triggers the drainage of the intervening air layer between it and the film. These results in the deformation of the drop shape and a radial spread of capillary ripples on the film surface, initiated at impact point (c-d). Once the air layer is ruptured, a merger of the liquid drop with the liquid film is observed (e) and a vertical growth of cylindrical liquid column (in a partial/total coalescence case). This is followed by a run-up of capillary waves on the column formed, which elongates it. Finally, a pinch-off of a satellite drop is observed (g-h), in a partial coalescence case, which is of smaller size to the initial mother drop. A repeat of the coalescence process is seen as well (i-j). Qualitative differences are seen in the outcomes observed (either bouncing or sliding or partial coalescence) and the presence of a cascade; while quantitative differences are observed in the pinch-off time, the size (height and width) of the liquid column formed, size of ejected satellite drop, and the cascade points. Please click here to view a larger version of this figure.

**Figure:**
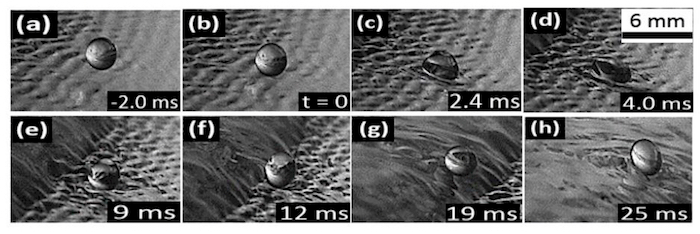

**Figure 5: Droplet sliding on the capillary wave region of a controlled flowing film.** The droplet diameter is 2.3 mm, with a fall height of 0.008 m while the film flow rate is 10 x 10^-3^ m^3^/s, corresponding to *Oh* = 0.0024, *We_d_* = 5.014, and film *Re *= 333, respectively. Forcing was carried out at 2 Hz. (a) Approach. (b) Contact. (c-f) Rolling drop. (g-h) Climbing the oncoming solitary hump. Please click here to view a larger version of this figure.

**Figure:**
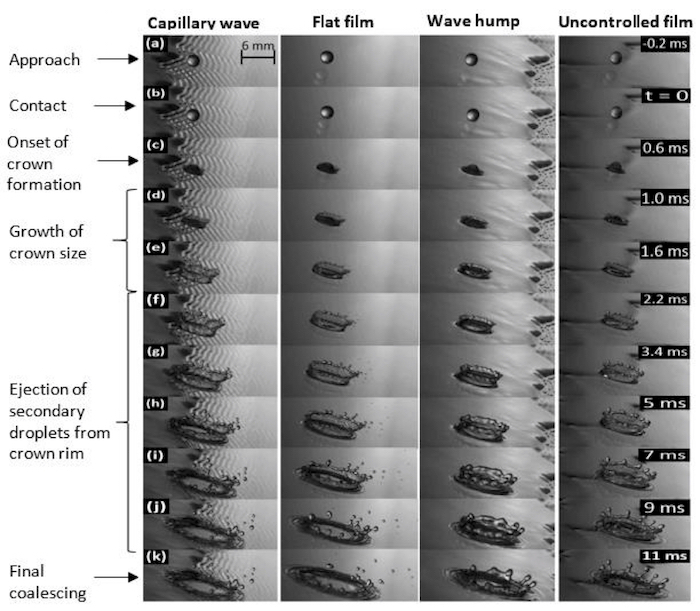

**Figure 6: Effect of film control on splashing phenomena on the different regions of impact on a controlled flowing film, contrasted against an uncontrolled film.** The droplet diameter is 3.3 mm, with a fall height of 0.25 m while the film flow rate is 5 x 10^-3^ m^3^/s, corresponding to *Oh* = 0.0021, *We_d _*= 224.8, and film *Re *= 166.5, respectively. Forcing was carried out at 2 Hz. The liquid drop approaches the film surface (a) and immediately upon contact (b), develops an ejecta sheet which grows into a crown (c). The growing crown (d-e) later yields to a Rayleigh-Plateau instability which leads to the ejection of smaller droplets from its rim (f-j). The crown collapses afterward and coalesces with the film (k), being transported away by the oncoming flow. The unique differences in the impact outcome on the individual regions of impact are seen in the size (height and diameter) of the crown formed, number and size distribution of ejected secondary drops, the degree of crown tilt, wall thickness, crown facing direction and final coalescence time. Reproduced from Adebayo & Matar 2017^15^ by permission of The Royal Society of Chemistry. Please click here to view a larger version of this figure.

**Figure:**
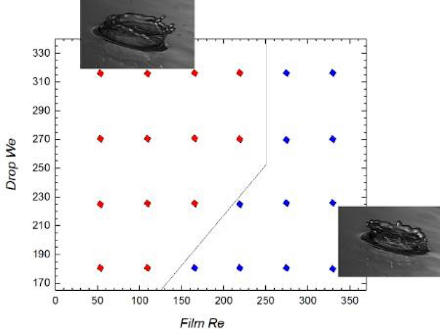

**Figure 7: Effect of film Reynolds and drop Weber on crown propagation in the 'wave hump region'.** The droplet size is 3.3 mm, corresponding to *Oh* = 0.0021 and the drop fall heights was varied from 0.20 to 0.35 m (corresponding to* We_d_* = 179.8 - 314.7) while *Re *is in the range of 55.5 to 333. The red diamonds depict outcomes with the crown facing the downstream direction while the blue diamonds show upstream-facing crown outcomes. The crown inclination is affected by the competition between the inertia of the impacting drop and that of the flowing film. Specifically, at low *Re*, the crown is inclined towards the streamwise direction but as the inertia of the flowing film gains in importance, the direction changes and faces upstream. This crown-upstream-facing direction is maintained beyond a *Re* value of approximately 250 regardless of the magnitude of *We_d_*. Reproduced from Adebayo & Matar 2017^15^ by permission of The Royal Society of Chemistry. Please click here to view a larger version of this figure.

**Figure:**
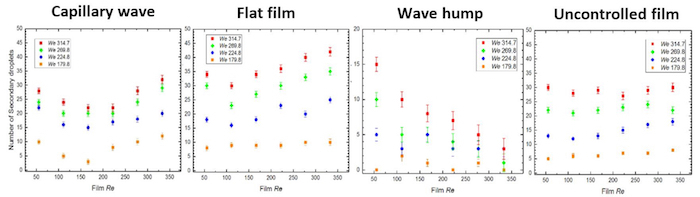

**Figure 8: Variation of the number of secondary droplets ejected from the crown rim in the different impact regions of a controlled film (viz 'capillary wave', 'flat film', and 'wave hump' regions, shown from left to right, respectively) contrasted against an uncontrolled film.** The droplet size is 3.3 mm corresponding to *Oh* = 0.0021, and the drop heights have been varied from 0.20 to 0.35, resulting in impact velocities within the range 1.981 - 2.621 m/s (corresponding to *We_d_* = 179.8 - 314.7). The red rectangles depict drop fall height of 0.35 m, the green diamonds 0.3 m, the blue circles 0.25 m, and the orange squares 0.2 m, respectively. The number of ejected secondary drops increase with drop *We* in all regions while an uneven trend is observed with film *Re* increase: On the wave hump, there is a decrease in the number of ejected secondary drops while on both capillary wave and flat film regions, there is a slight increase. A dip is noticed around film *Re* 166.5 for the capillary wave, which occurs as a result of the competition between the tangential velocities of the drop and that of the film. The disproportionate trend observed on the uncontrolled films is believed to occur as a result of the stochastic nature of the waves on the film surface. Reproduced from Adebayo & Matar 2017^15^ by permission of The Royal Society of Chemistry. Please click here to view a larger version of this figure.

**Figure:**
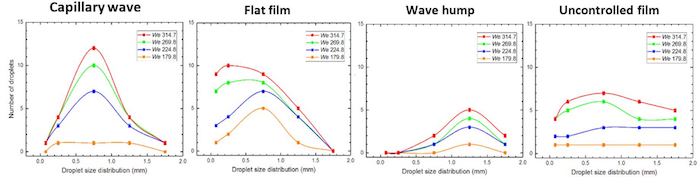

**Figure 9: Effect of impact region on the size distribution of ejected secondary droplet on a controlled film contrasted against an uncontrolled film.** The drop size is 3.3 mm while the film flow rate is 5 x 10^-3 ^m^3^/s corresponding to a film *Re* of 166.5 and drop *Oh *0.0021. The drop's fall heights are 0.2, 0.25, 0.3 and 0.35 m corresponding to *We_d_* 179.8, 224.8, 269.8 and 314.7 respectively. On the capillary wave, the shape of the distribution is largely unaltered with Weber number increase but a noticeable increase in the number of drops of the range 0.5 to 1.0 mm. On the flat films, however, the size distribution is observed to vary from 0 to 2.0 mm, and a shift is observed towards the 0 to 0.5 mm sized drops as the Weber number is increased. This increase in the number of small drops ejected clearly differentiates the flat film region from the other regions. On the wave hump, the size distribution shows that large drops in the range (1.0 to 2.0 mm) are ejected even for the smallest Weber number examined. In contrast to the above, the drop size distributions associated with an uncontrolled film do not exhibit a discernibly distinct shape owing to the stochastic nature of waves on such films. Reproduced from Adebayo & Matar 2017^15^ by permission of The Royal Society of Chemistry. Please click here to view a larger version of this figure.

**Table:** 

**Parameters **	**Capillary wave region**	**Flat film region**	**Wave hump region**
Apex height of liquid column	Short	Medium	High
Size of satellite drop	Small	Average	Large
Cascade existence	Rare	Yes	None
Effect of *Re* increase	Sliding phenomena	Bouncing phenomena	Transition to total coalescence
Effect of *We* increase	Decrease in column height	Increase in column height	Increase in column height
Effect of *Oh *decrease	Reduced drop sliding	Longer and wider columns, bigger satellite drops	Transition to total coalescence

**Table 1. **Parametric differences on low-inertia droplet impact dynamics on different regions of a controlled flowing film.

**Table:** 

**Parameters **	**Capillary wave region**	**Flat film region**	**Wave hump region**
Crown shape	Irregular	Irregular	Regular
Crown height	High	Higher	Highest
Crown wall thickness	Thin	Thinner	Thick
Number of secondary drops	More	Most	Little/None
Crown tilt angle	Reduces with film *Re*	Increases with film *Re*	Reverses beyond *Re* 250
Coalescing time	Quick	Slow	More delayed
Effect of film *Re *increase	Crown becomes more “upright”	Increase in crown height, steeper crown-inclination in film flow direction,	Decrease in number of secondary drops, change in crown-facing direction beyond *Re* 250
Effect of drop Weber increase	Earlier onset and increase in number of secondary drops, and increase in crown diameter.	Increase in number of secondary drops, crown height, and crown diameter; decrease in size of secondary drops	Increase in number of secondary drops, crown height, crown diameter, coalescence time, and change in crown-facing direction.
Effect of drop *Oh *decrease	Increase in crown diameter and height	Increase in crown diameter and height	Increase in crown diameter and height

**Table 2.** Parametric differences on high-inertia droplet impact dynamics on different regions of a controlled flowing film (the splashing regime).

## Discussion

In this section, we provide a few tips necessary to ensure qualitative results are obtained from the protocol. First, the glass substrate on which the liquid film flows must be kept completely dirt-free to ensure the properties of the liquid film are kept uncompromised. This is achievable by regular cleaning (probably using a suitable detergent, and wiped off over a tray to avoid dissolution into the system). Similarly, there should be a regular replacement of the whole test-liquid after some experimental rounds, to guarantee accurate results.

Secondly, the fluid-distribution chamber must be well-meshed and also kept air-tight to ensure the outflowing liquid film is uniform. This can be done by manually siphoning air out of the distribution box before each experiment. The use of micrometer-steps at the film inlet is also advised to set the gap-height at the film inlet to the exact film thickness predicted by the Nusselt estimate of the film flow at the corresponding Reynolds number. This will prevent a hydraulic jump or backflow at the inlet.

The operation of the solenoid valve must also always be checked and ascertained properly. This is because an appropriate pulsation of the flow is required to ensure the production of the forced waves. This could be checked from the regular clicking sound of the solenoid valve as well as a perceived pulsation along the connection pipes. The liquid flow rate into the syringe pump must also be set carefully to ensure the droplets are ejected in a dripping manner, avoiding any pre-acceleration before falling.

Appropriate calibration of the high-speed camera must be ensured to obtain very accurate results. The aperture size must also be carefully chosen considering parameters like the depth of field, exposure time and overall image brightness. For the camera triggering during video recording, users are also required to estimate how many frames should be recorded before triggering. This may vary with individuals, depending on the drop impact time, hence, several trial tests for practicing are recommended before actual measurements. Similarly, the light source must be properly arranged and well-diffused to minimize shadows in the image.

It is important to note and remember that the main focus of the study is the contributions of waves to the impact dynamics of the falling drops, hence the formation of regular wave structures is essential to an accurate study of the underlying physics. In scenarios where the wave structures are observed to quickly transition to three-dimensional structures, it is advised that the substrate inclination angle be reduced^14^,^19^ to facilitate a slower transition of the wave structures.

One limitation of the technique is observed in the absence of a measuring device specifying the actual instantaneous film thickness on each region of impact. This would have provided additional details on the overall observed phenomena.

In summary, the procedure outlined in this report can also be used to study simple wave evolution dynamics, while the high-speed imaging system described can be applied to many research fields with fast dynamics such as liquid drop break-up^21^,^22^/coalescence^23^, granular jets^24^, *etc.* where important phenomena are observed at a micro timescale.

## Disclosures

The authors have nothing to declare.
